# Reciprocal polyhedra and the Euler relationship: cage hydrocarbons, C*_n_*H*_n_* and *closo*-boranes [B*_x_*H*_x_*]^2−^

**DOI:** 10.3762/bjoc.7.30

**Published:** 2011-02-18

**Authors:** Michael J McGlinchey, Henning Hopf

**Affiliations:** 1 School of Chemistry and Chemical Biology, University College Dublin, Belfield, Dublin 4, Ireland; 2 Institut für Organische Chemie, Technische Universitaet Carolo-Wilhelmina zu Braunschweig, Hagenring 30, D-38106 Braunschweig, Germany

**Keywords:** cage hydrocarbons, high symmetry, reciprocal polyhedra

## Abstract

The *closo*-boranes B*_x_*H*_x_*_+2_, or their corresponding anions [B*_x_*H*_x_*]^2−^ (where *x* = 5 through 12) and polycycloalkanes C*_n_*H*_n_* (where *n* represents even numbers from 6 through 20) exhibit a complementary relationship whereby the structures of the corresponding molecules, e.g., [B_6_H_6_]^2−^ and C_8_H_8_ (cubane), are based on reciprocal polyhedra. The vertices in the *closo*-boranes correspond to faces in its polycyclic hydrocarbon counterpart and vice versa. The different bonding patterns in the two series are described. Several of these hydrocarbons (cubane, pentagonal dodecahedrane and the trigonal and pentagonal prismanes) are known while others still remain elusive. Synthetic routes to the currently known C*_n_*H*_n_* highly symmetrical polyhedral species are briefly summarized and potential routes to those currently unknown are discussed. Finally, the syntheses of the heavier element analogues of cubane and the prismanes are described.

## Review

### Platonic polyhedra and the Euler relationship

**Figure 1:**
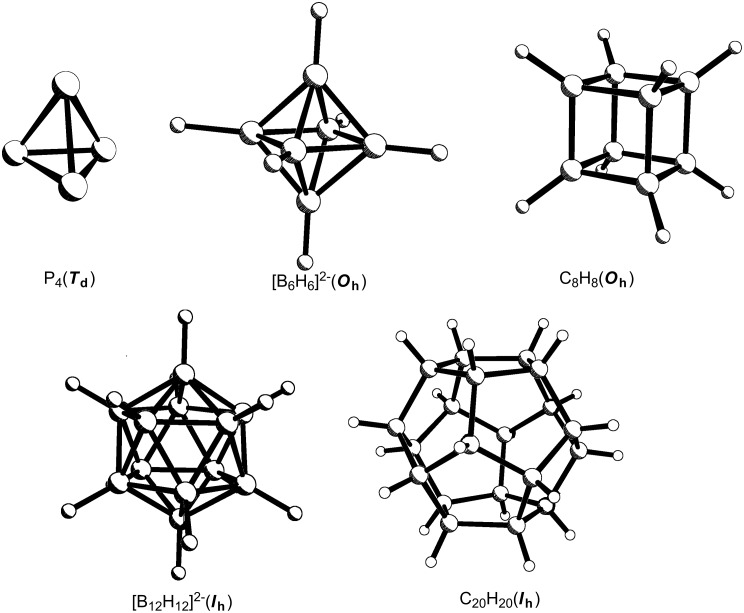
Molecular analogues of the Platonic solids.

The Platonic solids have long fascinated geometers, artists and chemists alike. Molecular analogues of the tetrahedron (P_4_, B_4_Cl_4_, Si_4_*t-*Bu_4_), octahedron ([B_6_H_6_]^2−^), cube (C_8_H_8_), icosahedron ([B_12_H_12_]^2−^) and pentagonal dodecahedron (C_20_H_20_) are now known (Figure 1). The tetravalency of carbon makes the C*_n_*H*_n_* molecules viable only for the tetrahedron, cube and dodecahedron.

**Figure 2:**
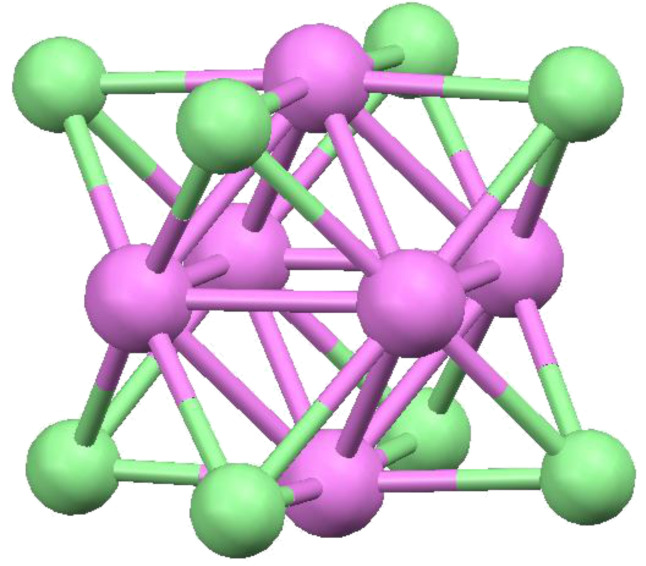
The structure of [Mo_6_Cl_8_]^4+^ demonstrates the reciprocal relationship between the cube and the octahedron.

It has been recognized for millennia that there is a simple relationship between pairs of Platonic solids [1]. If the centers of adjacent faces of the octahedron are connected, they yield a cube and vice versa; this is beautifully illustrated by the X-ray crystal structure of the [Mo_6_Cl_8_]^4+^ cluster in which an octahedron of molybdenum atoms is encapsulated within a cube of chlorines (Figure 2). The icosahedron and pentagonal dodecahedron are similarly related; these pairs of "reciprocal" or "dual" polyhedra possess the same point group symmetry [2].

As noted by René Descartes around 1620 and stated formally by Leonhard Euler in 1752, for any convex polyhedron there is a simple relationship between the number of vertices (***V***), faces (***F***) and edges (***E***):

***V*** + ***F*** = ***E*** + 2

Thus, the cube has 8 vertices, 12 edges and 6 faces; its reciprocal polyhedron – the octahedron – possesses 6 vertices, 12 edges and 8 faces. Likewise, the ***V***, ***E*** and ***F*** values for the icosahedron (12, 30, 20) and pentagonal dodecahedron (20, 30, 12) are in accord with Euler's equation. Interestingly, the tetrahedron (4 vertices, 6 edges and 4 faces) is its own reciprocal.

### Boranes, hydrocarbons and inverse polyhedra

**Figure 3:**
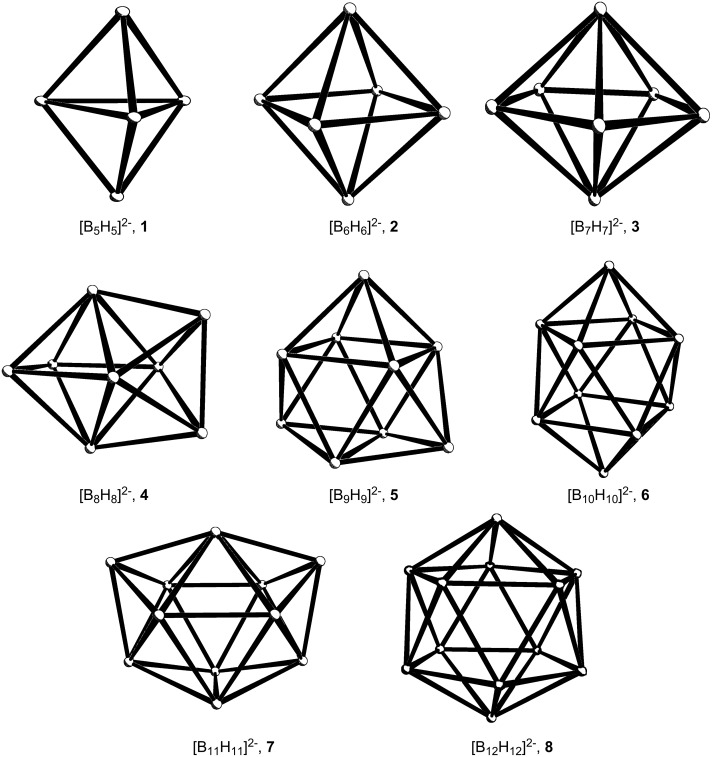
The deltahedra corresponding to the structures of the *closo*-boranes [B*_x_*H*_x_*]^2−^.

*Closo*-borane anions and their carborane analogues adopt polyhedral structures in which each face is triangular [3]; Figure 3 shows the deltahedra corresponding to the [B*_x_*H*_x_*]^2−^, **1**–**8**, (*x* = 5 through 12) or C_2_B*_x_*_-2_H*_x_* series and Table 1 lists their point groups and ***V***, ***E*** and ***F*** values. (One must emphasize that in these formally electron-deficient systems, the edges do not represent two-electron bonds but merely indicate the structure). Now, every deltahedron has a reciprocal polyhedron in which each triangular face has become a vertex linked to three neighbors; this is precisely the criterion that has to be satisfied by alkanes of the C*_n_*H*_n_* type.

**Table 1:** Corresponding *closo*-boranes and polycycloalkanes of the same symmetry.

*closo*-borane		***V***	***E***	***F***	point group	***V***	***E***	***F***	polycycloalkane	

[B_5_H_5_]^2−^	**1**	5	*9*	6	*D*_3_*_h_*	6	*9*	5	C_6_H_6_	[3]prismane **9**
[B_6_H_6_]^2−^	**2**	6	*12*	8	*O_h_*	8	*12*	6	C_8_H_8_	[4]prismane (cubane) **10**
[B_7_H_7_]^2−^	**3**	7	*15*	10	*D*_5_*_h_*	10	*15*	7	C_10_H_10_	[5]prismane (pentaprismane) **11**
[B_8_H_8_]^2−^	**4**	8	*18*	12	*D*_2_*_d_*	12	*18*	8	C_12_H_12_	[4^4^.5^4^]octahedrane **12**
[B_9_H_9_]^2−^	**5**	9	*21*	14	*D*_3_*_h_*	14	*21*	9	C_14_H_14_	[4^3^.5^6^]nonahedrane **13**
[B_10_H_10_]^2−^	**6**	10	*24*	16	*D*_4_*_d_*	16	*24*	10	C_16_H_16_	[4^2^.5^8^]decahedrane **14**
[B_11_H_11_]^2−^	**7**	11	*27*	18	*C*_2_*_v_*	18	*27*	11	C_18_H_18_	[4^2^.5^8^.6]undecahedrane **15**
[B_12_H_12_]^2−^	**8**	12	*30*	20	*I_h_*	20	*30*	12	C_20_H_20_	[5^12^]dodecahedrane **16**

To illustrate this inverse polyhedral relationship between *closo*-boranes, [B*_x_*H*_x_*]^2−^ **1**–**8**, and C*_n_*H*_n_* hydrocarbons (*n* = 6, 8, 10 … 20; **9**–**16**), the point groups and ***V***, ***E***, ***F*** values of the complementary C*_n_*H*_n_* molecules are collected in Table 1.

### Bonding comparisons

The inverse geometric structures of the *closo*-boranes and their cage hydrocarbon complementary counterparts are the result of the different electronic configurations of boron and carbon. The C*_n_*H*_n_* systems are assembled from CH units each of which supplies three atomic orbitals and three electrons to the cage. Each carbon can link to three others via conventional two-electron bonds, thus forming electron-precise molecules. In contrast, BH units also provide three atomic orbitals but only two electrons for cage bonding; as a result, the *closo*-boranes are electron-deficient molecules with skeletal connectivities greater than three. Their total number of skeletal electron pairs equals the number of vertices plus one; for example, [B_6_H_6_]^2−^ has 7 skeletal electron pairs and is three-dimensionally aromatic. In contrast, in the C*_n_*H*_n_* cages the number of skeletal electron pairs equals the number of edges. In terms of the Euler equation (***V*** + ***F*** = ***E*** + 2), for cage hydrocarbons, 2***E*** = 3***V***, as exemplified by cubane, C_8_H_8_, which has 12 edges and 8 vertices, whereas for *closo*-boranes it is evident that 2***E*** = 3***F***, as in [B_8_H_8_]^2−^ which has 18 edges and 12 faces.

However, one must not assume that bonds are fragile in molecules for which the ratio of valence electrons to interatomic linkages is less than two. For example, the carborane 1,12-B_10_C_2_H_12_ (an icosahedral molecule in which the carbons are maximally separated) only suffers serious decomposition at 630 °C [4], a temperature very much higher than that at which the vast majority of electron-precise organic molecules would survive.

The existence of a complete set of *closo*-boranes, B*_x_*H*_x_*_+2_, or their corresponding anions [B*_x_*H*_x_*]^2−^, where *x* = 5 through 12, suggests that their complementary hydrocarbon cages C*_n_*H*_n_*, where *n* represents the even numbers 4 through 20, should also all be viable, as discussed herein.

### Synthetic routes to highly symmetrical polycyclic hydrocarbons

As already noted, molecular analogues of the Platonic solids are known, and the first syntheses of cubane, **10**, (by Philip Eaton) [5], and of pentagonal dodecahedrane, **16**, (by Leo Paquette) [6–7] are now classics. [3]Prismane, C_6_H_6_, **9**, (by Tom Katz) and [5]prismane, C_10_H_10_, **11**, (also by Eaton) have been reported, but the remaining hydrocarbons listed in Table 1 still pose serious synthetic challenges. Here, we briefly summarize the successful routes to **9**, **10**, **11** and **16**, include selected publications on the synthesis of derivatives of tetrahedrane, discuss the current status of some "polycycloalkane near misses", and suggest that a *C*_2_*_v_* isomer of C_18_H_18_, **15**, should be a worthwhile synthetic target.

#### Tetrahedrane, C_4_H_4_

**Scheme 1:**
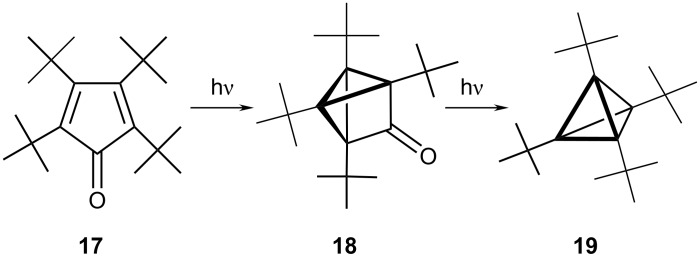
The first synthesis of a tetrahedrane **19** by Maier.

The search for tetrahedrane has a long history [8] and the parent molecule still resists isolation. However, Maier and co-workers were able to prepare the tetra-*tert*-butyl derivative, **19**, as the first known derivative of this simplest of the Platonic bodies by photolysis of tetra-*tert*-butylcyclopentadienone, (**17**). As depicted in Scheme 1, the initial "criss-cross" product, **18**, eventually loses CO to yield **19**, as a stable crystalline material [9]. Presumably, in addition to the unfavorable electronic factors associated with cyclobutadienes, steric interactions between the bulky alkyl groups destabilize the planar system. However, the formation of a molecule containing four cyclopropyl moieties clearly introduces considerable additional ring strain.

#### [3]Prismane, C_6_H_6_, **9**

**Scheme 2:**
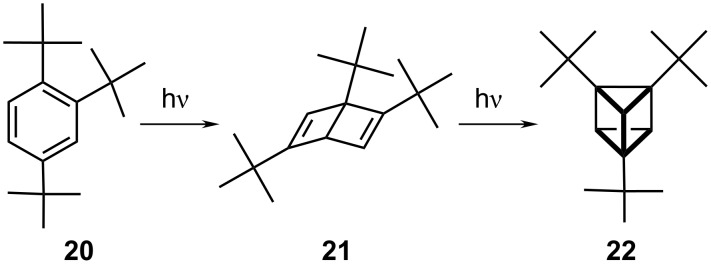
The conversion of Dewar benzenes to [3]-prismanes.

Prismane derivatives bearing bulky substituents (e.g., *t-*Bu, CF_3_, Ph; **20**) have been available for more than three decades via photolysis of sterically encumbered benzenes [10]. The marked deviation from planarity in these systems favors the formation of Dewar benzenes, **21**, which undergo [2 + 2] cycloadditions to produce the prismane skeleton, **22**, (Scheme 2).

**Scheme 3:**
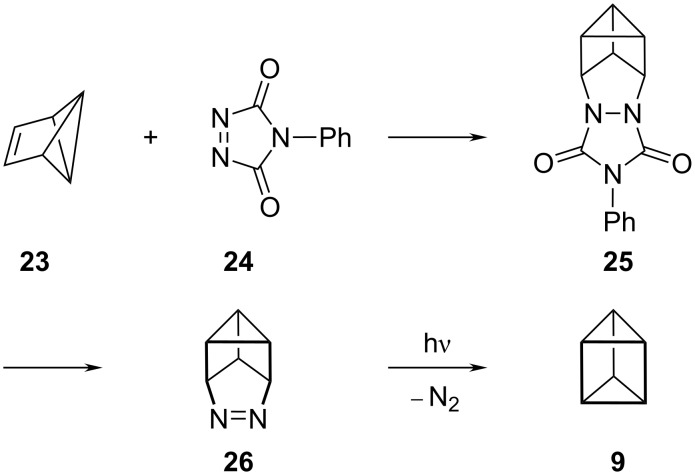
Synthesis of [3]prismane **9** by Katz.

However, the parent prismane, **9**, proved much more elusive and was finally obtained in 2% yield by treatment of benzvalene, (**23**), with *N*-phenyltriazolindione, (**24**), to give cycloadduct **25**; conversion to the azo compound **26** and photolysis to extrude nitrogen finally led to **9** [11] (Scheme 3). Although the yield of the final photolysis step has now been improved somewhat to 15% [12], [3]prismane is still not a conveniently obtainable molecule.

#### Cubane, C_8_H_8_, **10**

**Scheme 4:**
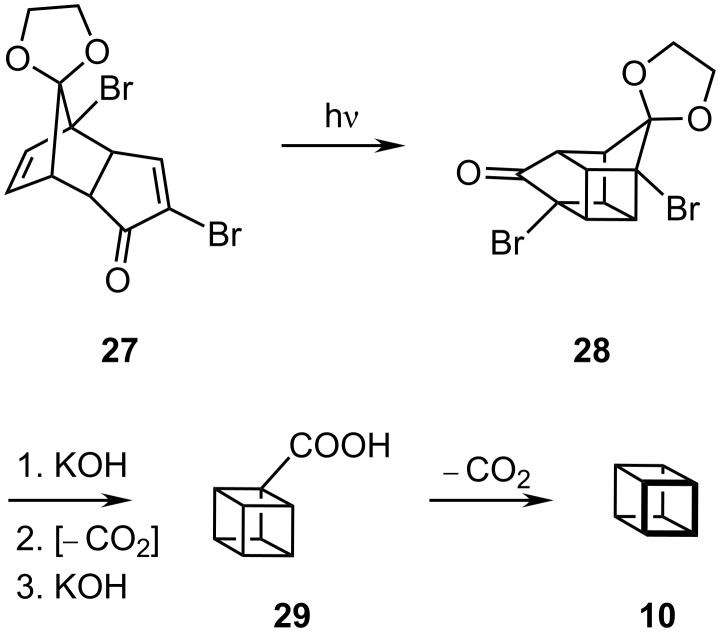
Synthesis of cubane **10** by Eaton.

The key step of Eaton's beautiful synthesis of [4]prismane (cubane, **10**), shown in Scheme 4, involves the ring contraction of the mono-protected diketone **28**, itself available via photolysis of **27**, the mono-ketal of the Diels–Alder dimer of 2-bromocyclopentadienone. At this point, the bishomocubanedione system is exquisitely poised to undergo successive Favorskii reactions to yield eventually the carboxylic acid **29**, which furnishes cubane upon decarboxylation [5].

**Scheme 5:**
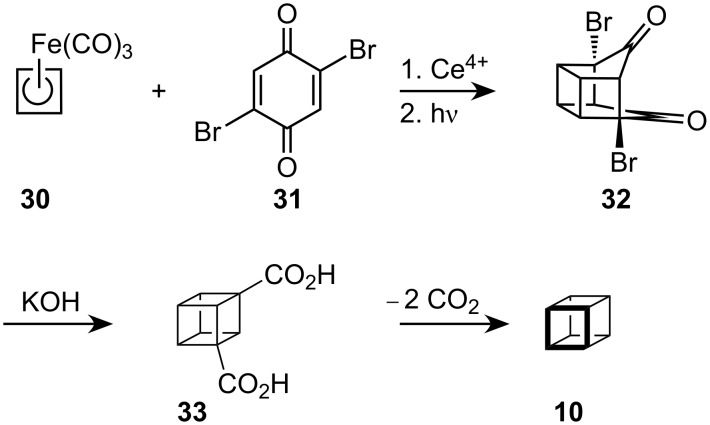
Synthesis of cubane **10** by Pettit.

An elegant modification of this procedure has been reported by Pettit [13] who used (cyclobutadiene)Fe(CO)_3_, (**30**), as the source of one of the square faces (Scheme 5). Once again, as in Eaton's procedure, two Favorski ring contractions were used to obtain the cubane skeleton, first in the form of the dicarboxylic acid **33** which on subsequent decarboxylation gave **10**.

The original approach has since been considerably improved and modified. Now, functionalized cubanes can be obtained in kilogram quantities and their chemistry has been extensively studied. For example, polynitrocubanes have been investigated as high-energy-density materials [14–15] and the cardiopharmacological activity of cubane dicarboxylic acid and its amide has been reported [16].

#### [5]Prismane, C_10_H_10_, **11**

**Scheme 6:**
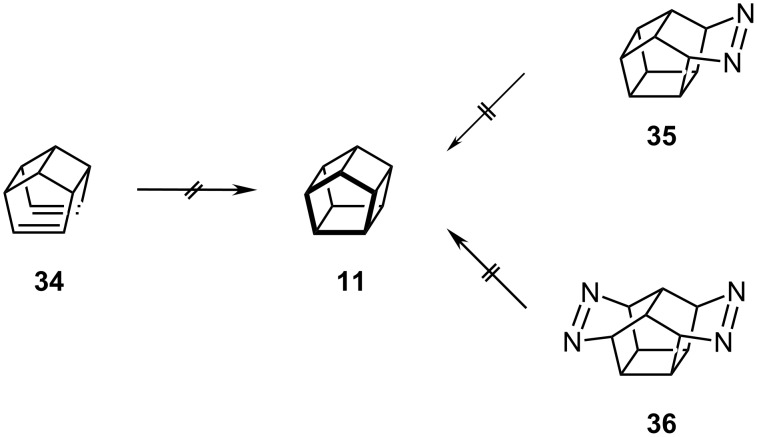
Failed routes to [5]-prismane **11**.

Since pentaprismane, **11**, is the least strained of the prismanes, one might have expected it to be readily available by photolytic [2 + 2] cycloaddition of hypostrophene, **34**, or by extrusion of nitrogen from either **35** or **36**, as in Scheme 6 [17–20]; surprisingly, all these routes were found to be ineffective.

**Scheme 7:**
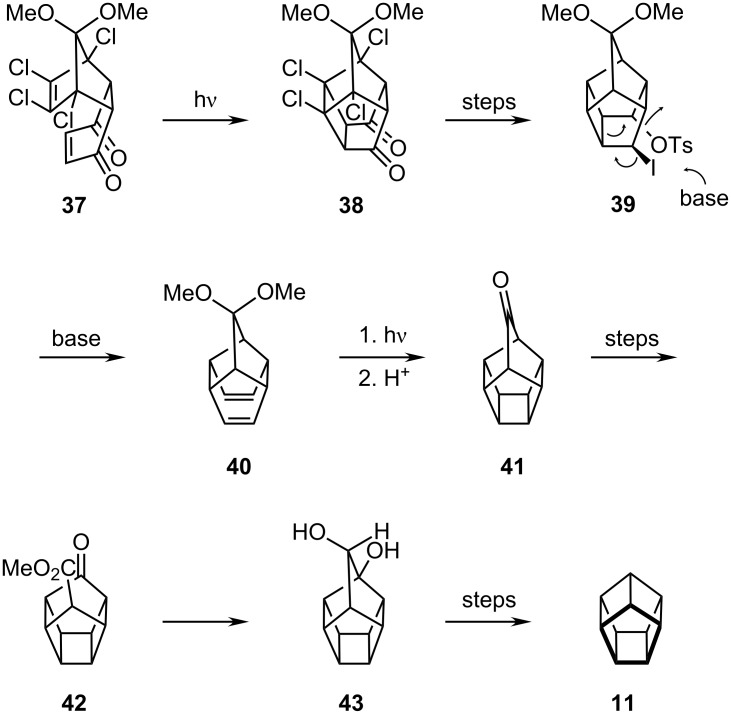
Synthesis of [5]prismane **11** by Eaton.

Success was finally achieved via ring contraction of a homopentaprismane, somewhat analogous to the original cubane synthesis. As shown in Scheme 7, Diels–Alder reaction of 1,2,3,4-tetrachloro-5,5-dimethoxycyclopentadiene with *p*-benzoquinone gave **37** which underwent photolytic [2 + 2] closure to the pentacyclic dione **38**. Subsequent dechlorination and functional group manipulation led to the iodo-tosylate **39** which, in the presence of base, generated the homohypostrophene, **40**; [2 + 2] cycloaddition then furnished the homopentaprismanone **41**. Introduction of a bridge head bromine (with the intent of carrying out a Favorskii ring contraction) proved to be impossible. Instead it was necessary to proceed via the keto-ester **42** and the dihydroxyhomopentaprismane **43** which, after ring contraction and decarboxylation, yielded **11** [21–22].

#### Pentagonal dodecahedrane, C_20_H_20_, **16**

The preparation of dodecahedrane, **16**, is undoubtedly one of the great synthetic achievements of recent times and will remain at the forefront of alicyclic chemistry until a stepwise synthesis of C_60_ is achieved. However, we note en passant that Scott, de Meijere and their colleagues have devised a rational route to C_60_ from a chlorinated precursor, C_60_H_27_Cl_3_, in which only the final ring closures were achieved via preparative-scale flash vacuum pyrolysis [23].

**Scheme 8:**
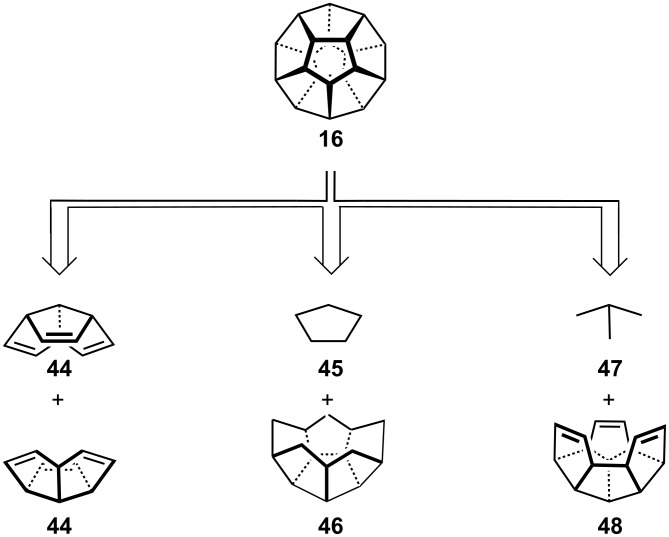
Retrosynthetic analysis for several approaches to dodecahedrane **16**.

The icosahedral point group possesses ten *C*_3_ axes and six *C*_5_ axes, and synthetic proposals have taken advantage of both types of symmetry elements. The former prompted Woodward [24] and Jacobson [25] independently from each other to suggest that two triquinacene units could be coupled (Scheme 8). The requisite C_10_H_10_ moiety, **44**, has been prepared (most elegantly via Paquette’s domino Diels–Alder route [26]) but all attempts at controlled dimerization (even on a transition metal template [27]) have so far proven fruitless. A second approach is based upon the five-fold symmetry of [5]peristylane, **46**; this system has been accessed by Eaton (Scheme 8) but attempts to add the 5-carbon roof, **45**, have not yet succeeded [28]. Another "3-fold" approach relies on the addition of a trimethylenemethane-like C_4_ fragment, **47**, to C_16_-hexaquinacene, **48**; but again complications arose during attempts to convert this molecule to **16** [29].

**Scheme 9:**
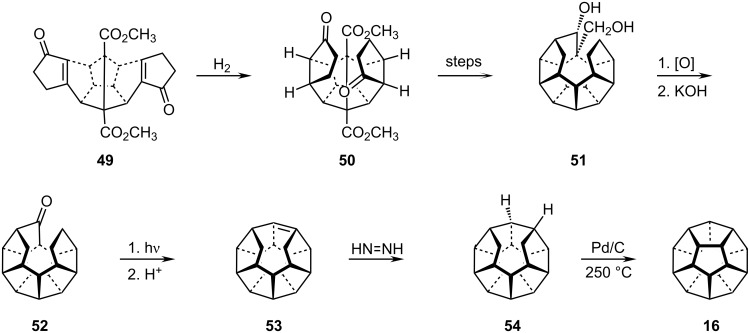
Paquette´s synthesis of dodecahedrane **16**.

As outlined in Paquette's eloquent overview of the history of the dodecahedrane project [30], success was finally achieved via the C_2_ route summarized in Scheme 9. The crucial intermediate, **49**, was hydrogenated to **50**, which was converted in several cyclization steps to the diol **51**. Oxidation and condensation of the resulting ketoaldehyde then provided the mono ketone **52**, which was photochemically ring-closed to the secododecahedrene **53**. After diimine hydrogenation to **54** only one C–C bond in the nearly completed sphere was lacking. This final cyclodehydrogenation to **16** was accomplished by treatment of **54** with Pt/C at 250 °C [31–32].

**Scheme 10:**
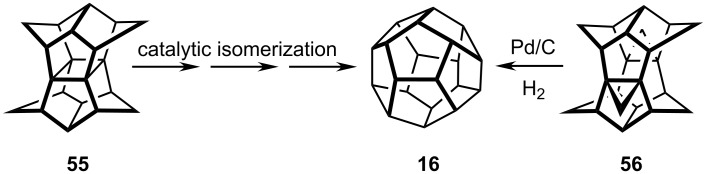
Prinzbach´s synthesis of dodecahedrane **16**.

Subsequently, Prinzbach established an entirely different route to dodecahedrane (Scheme 10) that proceeds by catalytic isomerization of pagodane, **55**, a molecule of *D*_2_*_h_* symmetry, which itself was prepared by a multi-step route from readily available starting materials. Alternatively, the bis-cyclopropanated hydrocarbon **56** yielded dodecahedrane on treatment with Pd/C in a hydrogen atmosphere. Once again, the reader is referred to Prinzbach's comprehensive review of his group's major contributions to this area [33].

### Currently unknown polyhedranes, C*_n_*H*_n_*, *n* = 12, 14, 16, 18

**Figure 4:**
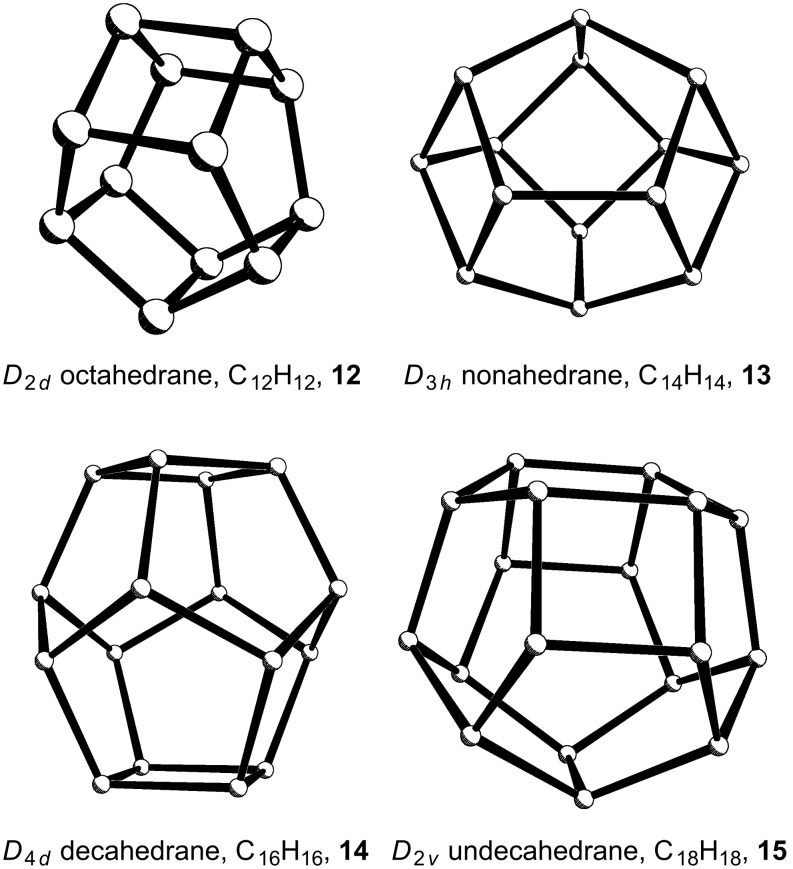
The as yet unknown polyhedranes **12**–**15**.

After this short overview of the successful syntheses of several polycyclic alkanes, C*_n_*H*_n_*, where *n* = 4, 6, 8, 10 and 20, we now turn to the remaining unknown polyhedranes for which *n* = 12 (octahedrane, **12**), *n* = 14 (nonahedrane, **13**), *n* = 16 (decahedrane, **14**), and *n* = 18 (undecahedrane, **15**) (Figure 4). Although none of these have as yet been reported, there is some beautiful chemistry associated with their potential precursors, and one can only admire the ingenuity of the talented investigators in this area.

#### Octahedrane, C_12_H_12_, **12**

**Figure 5:**
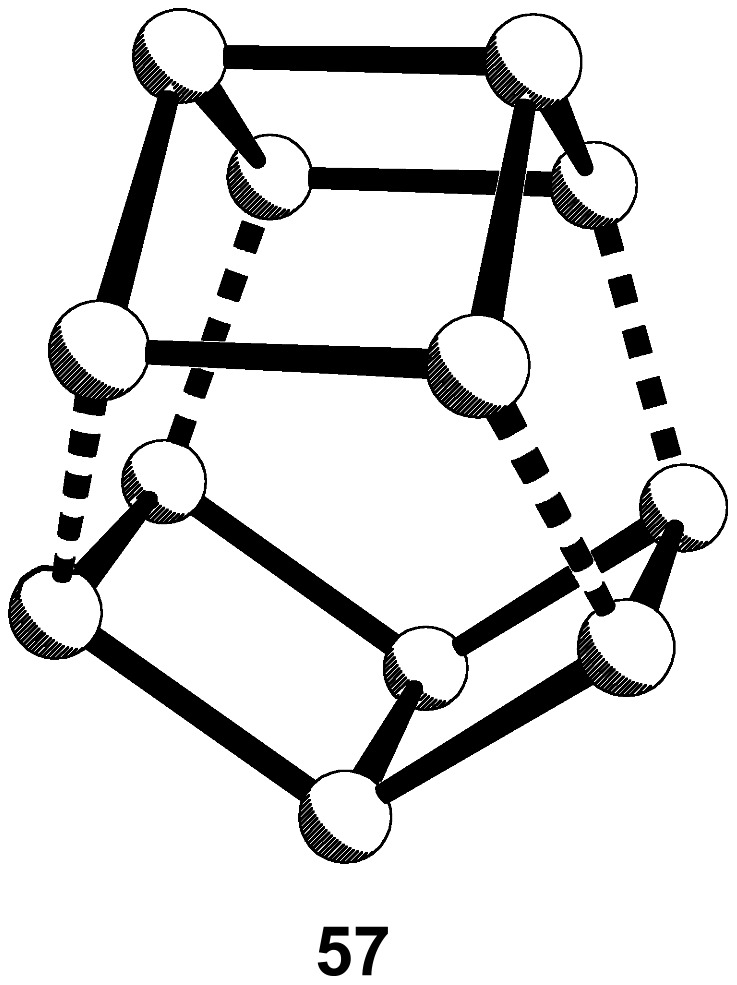
Coupling of two Dewar benzenes.

One might envisage a direct route to **12** by the coupling of two Dewar benzenes as in **57** (Figure 5), but we are unaware of any such reports.

**Scheme 11:**
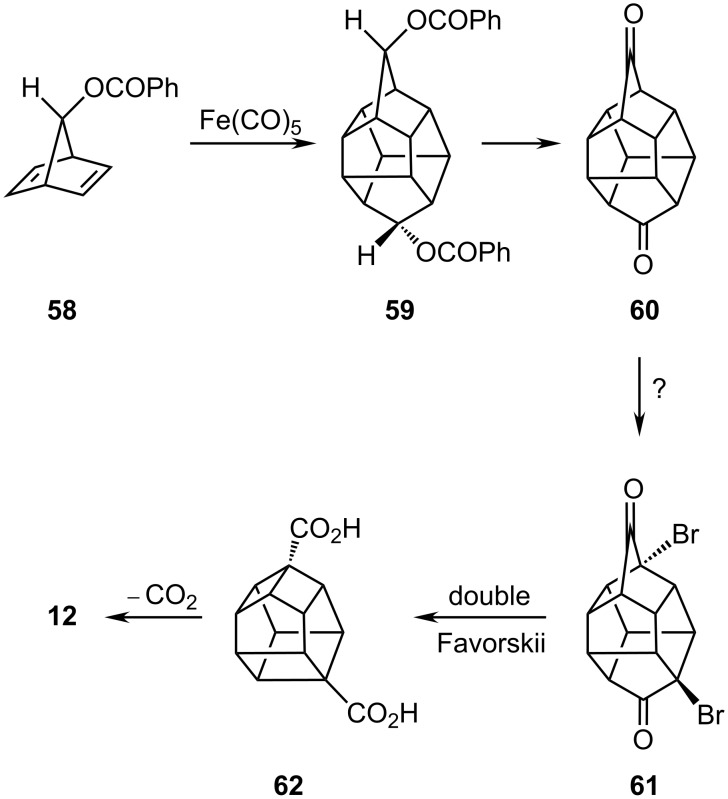
A possible route to octahedrane **12**.

However, metal carbonyl promoted dimerization of norbornadienes, e.g., **58** to **59**, is a well established protocol, and Marchand [34] has exploited this reaction to prepare the C_14_ diketone **60** (Scheme 11). In principle, one could incorporate bridgehead halogens as in **61** with the aim of carrying out two Favorskii ring contractions to generate the octahedrane skeleton, viz. the dicarboxylic acid **62**, which ultimately would then require to be decarboxylated to produce the parent system **12**. More realistically, one can see the obvious similarity to the conversion of homopenta-prismanone, **41**, to pentaprismane, **11**, which was successfully accomplished via Baeyer–Villiger oxidation, acyloin coupling and decarboxylation. Thus, one might anticipate that such an approach might provide access to *D*_2_*_d_* [4^4^.5^4^]octahedrane **12**. (We note that a simplified nomenclature has been proposed in which the number of 3, 4 or 5-membered rings is indicated by a superscript; thus cubane is [4^6^]hexahedrane and pentaprismane is [4^5^.5^2^]heptahedrane [35]).

#### Nonahedrane, C_14_H_14,_ **13**

**Scheme 12:**
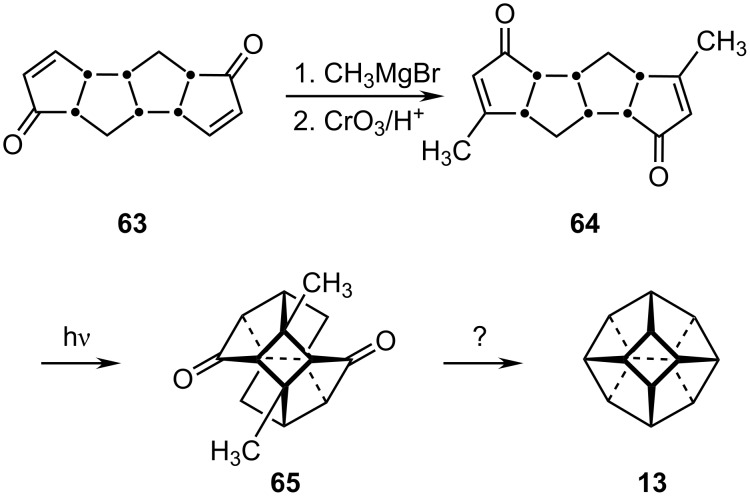
A possible route to nonahedrane **13**.

It has been proposed that an intermediate in the synthesis of [5]peristylane may be a viable precursor to the still unknown *D*_3_*_h_* [4^3^.5^6^]nonahedrane, (**13**). As shown in Scheme 12, the diene-dione **63** can be readily converted to the double enone **64** which undergoes [2 + 2] photocyclization to yield the pentacyclic diketone **65** [36]. We await further elaboration of this fascinating system.

#### Decahedrane, C_16_H_16,_ **14**

**Figure 6:**
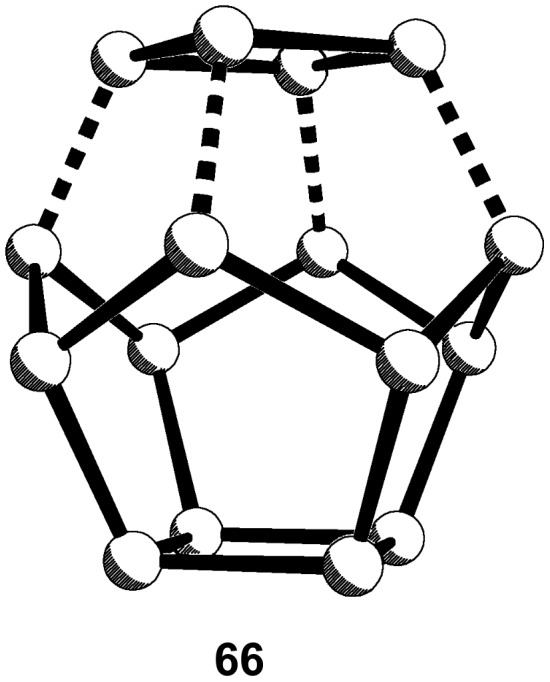
Capping [4]peristylane with a four-membered ring system.

Formally, **14** should be available by capping [4]peristylane with a four-membered ring system, as in **66** (Figure 6).

**Scheme 13:**
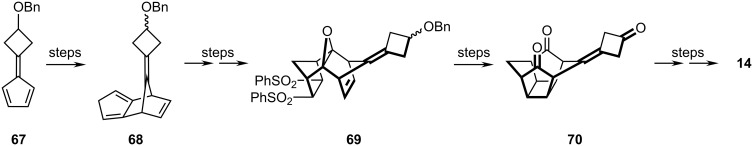
A possible route to decahedrane **14**.

However, to translate this concept into a preparatively realistic protocol is a different matter. Even if there is no report of a completed synthesis of **14**, considerable progress has been made [37]. The route taken by Paquette and co-workers (Scheme 13) required the initial generation of the fulvene **67**, whose four-membered ring should eventually serve as the "roof" of a [4]peristylane "building". Towards this goal, **67** was first converted into the cyclopentadiene derivative **68**, whose carbon skeleton was subsequently extended, and then bent into a convex shape by an epoxidation reaction (formation of **69**). After the still saturated C_2_-bridge had been reduced to an etheno bridge, the prerequisite for an intramolecular [2 + 2] photoaddition had been created. Indeed, photochemical ring closure and various oxidation steps led to the "open" triketone **70** that in principle should be convertible to a seco-decahedrane skeleton by two aldol condensations. This latter system should close to **14** by taking advantage of methodology established during the dodecahedrane project.

#### Undecahedrane, C_18_H_18,_ **15**

**Figure 7:**
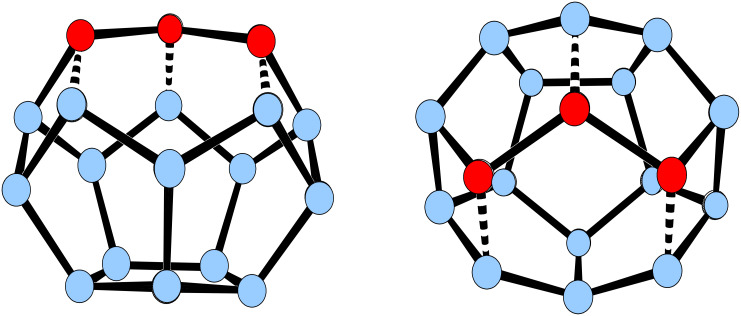
A possible route to undecahedrane **15** (left: side view; right: top view).

**Scheme 14:**
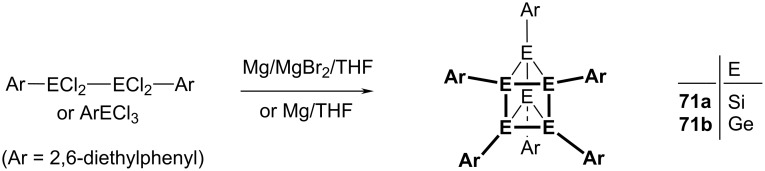
Synthetic routes to trigonal prismatic hexasilanes **71a** and hexagermanes **71b**.

The reciprocal polyhedron to the octadecahedron B_11_H_11_^2−^, **7**, is the *C*_2_*_v_* [4^2^.5^8^.6]undecahedrane, **15** (Figure 7). Note that this C_18_H_18_ system contains a 6-membered ring paralleling the *C*_2_*_v_* symmetry of the borane that has a capping boron linked to six others. We are unaware of any attempted syntheses of this molecule but, as depicted in Scheme 14, suitable disconnections reveal that a C_3_-bridged ansa-[5]peristylane is an enticing precursor to **15**.

### Highly symmetric inorganic polyhedranes

The major synthetic challenges that needed to be overcome to synthesize the polyhedranes [3]prismane, (**9**), cubane, **10**, and [5]prismane, **11**, contrast sharply with the ready availability of their heavier congeners. Thus, trigonal prismatic hexasilanes, **71a**, and hexagermanes, **71b**, are accessible in single-step processes by sodium- or magnesium-mediated dehalogenation of the appropriate R**E**X_3_ precursor, where R is a very bulky alkyl or aryl group, and X is chlorine or bromine [38–39]. The hexatellurium cation, [Te_6_]^4+^, is also trigonal prismatic [40–41].

**Scheme 15:**
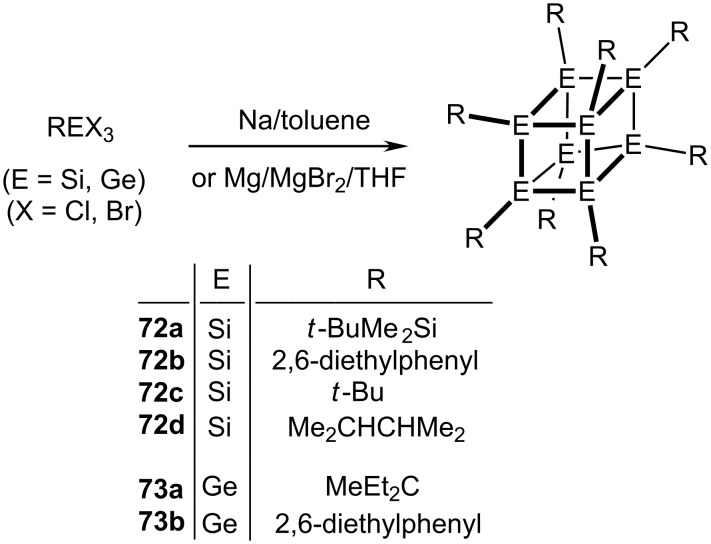
Synthetic routes to octasila- and octagerma-cubanes.

**Scheme 16:**
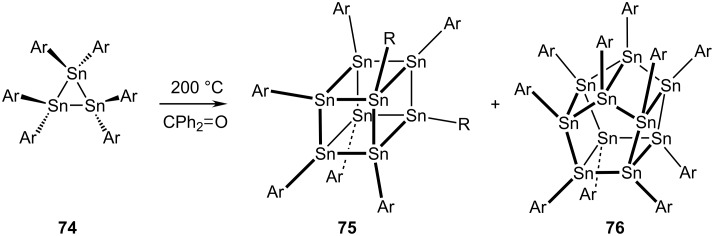
Synthesis of an octastannacubane and a decastannapentaprismane.

Similarly, the inorganic cubane analogues R_8_**E**_8_, where **E** = Si, **72**, and **E** = Ge, **73**, where R is again a bulky group, are also well-known (Scheme 15). Indeed, octakis(*t*-butyldimethylsilyl)octasilane, (**72a**), is obtained in 72% yield by treatment of the corresponding trichlorosilane precursor with sodium in toluene at 90 °C. These systems have been thoroughly investigated structurally and spectroscopically, and their reactivity has also been extensively investigated [42–45]. Furthermore, as shown in Scheme 16, the octastannacubane, **75**, and the per-arylated decastannane, **76**, a tin analogue of pentaprismane (**11**) have been prepared by thermolysis of hexakis(2,6-diethylphenyl)cyclotristannne, **74**, and fully characterized spectroscopically and by X-ray crystallography [46–48].

The major mitigating factor here is that such elements commonly form structures in which 90° angles are the norm [49], and so ring strain is no longer such a major impediment to bond formation. While the intrinsic yields of these products can range from very good to rather poor, this is compensated by the fact that they involve short syntheses from relatively inexpensive starting materials.

**Scheme 17:**
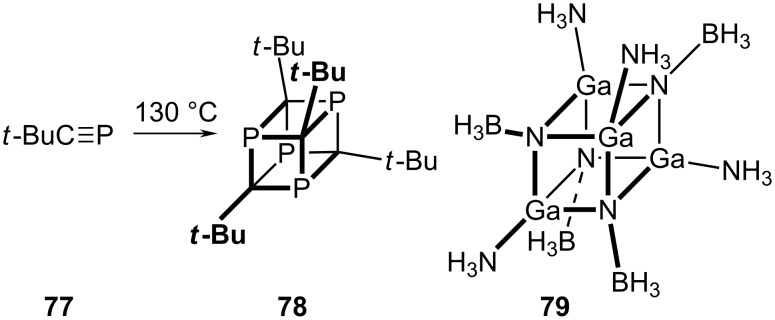
Synthesis of a heterocubane.

Another particularly interesting system is the mixed carbon-phosphorus heterocubane, **78**, prepared in 8% yield simply by heating the phosphaalkyne *t*-BuC≡P, **77**, at 130 °C for several days (Scheme 17) [50]. Finally, we note an interesting recent paper that reported high level theoretical calculations on the structures and stabilities of heterocubanes [XY]_4_ comprised of Group 13 (X = B, Al, Ga) – Group 15 (Y = N, P, As) tetramers [51]. It was shown that they should be stabilized when decorated with donor–acceptor linkages as in **79**. Moreover, it was suggested that they may function as single source precursors of Group 13 – Group 15 materials with applications in microelectronics.

## Conclusion

We have endeavored to illustrate the complementary relationship between *closo*-boranes [B*_x_*H*_x_*]^2−^, where *x* = 5 through 12, and polycycloalkanes C*_n_*H*_n_*, where *n* represents even numbers from 6 through 20. Several of these hydrocarbons are known while others remain elusive. Interestingly, one can invert the original concept and propose that other highly symmetrical cage hydrocarbons of the C*_n_*H*_n_* type might have *closo*-borane counterparts. Indeed, Lipscomb and Massa have discussed the structures of borane analogues of fullerenes [52–53] and even of nanotubes [54]. In particular, they proposed that C_60_ (***V***, ***E***, ***F*** = 60, 90, 32) could have a corresponding *closo*-borane [B_32_H_32_]^2−^ (***V***, ***E***, ***F*** = 32, 90, 60) of icosahedral symmetry [55].

**Figure 8:**
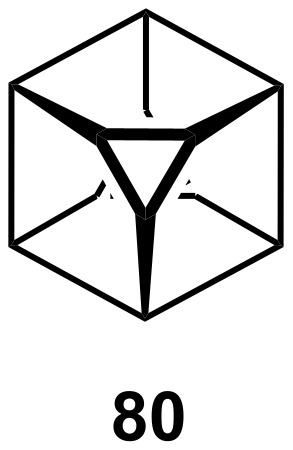
*D*_3_*_d_* symmetric C_8_H_8_, a bis-truncated cubane.

Similarly, in the C_8_H_8_ series, de Meijere [56] has prepared *D*_3_*_d_* [3^2^.5^6^]octahedrane, **80**, (Figure 8; ***V***, ***E***, ***F*** = 12, 18, 8), and one might envisage the existence of a comparable "electron deficient" molecule of bicapped octahedral symmetry (***V***, ***E***, ***F*** = 8, 18, 12); indeed, Muetterties noted that *D*_2_*_d_* [B_8_H_8_]^2−^ is highly fluxional and exists in equilibrium with several other isomers [57–58]. Moreover, transition metal clusters, such as [Re_8_C(CO)_24_]^2−^, provide examples of such a bicapped octahedral *D*_3_*_d_* geometry [59]. Interestingly, King et al. have pointed out the reciprocal polyhedral relationship between gold clusters and fullerenes [60].

As noted above, molecular analogues of several members of both sets of the complementary polyhedra exhibited by the *closo*-boranes and by the polycycloalkanes have been constructed from other elemental species: Clusters containing lithium, transition metals, silicon, phosphorus, arsenic, bismuth, lead, etc. have been characterized [61–64], and their architectures continue to delight us. The existence of molecules of such exquisite symmetry would surely have fascinated Plato.
